# A novel neuroimmune modulation system for the treatment of rheumatoid arthritis

**DOI:** 10.1186/s42234-024-00142-9

**Published:** 2024-04-03

**Authors:** Bruno Bonaz

**Affiliations:** Université Grenoble Alpes-Faculté de Médicine, Grenoble Institut Neurosciences (GIN, Inserm U1216), site Santé, Bâtiment Edmond J. Safra, 31 Chem. Fortuné Ferrini, 38700 La Tronche, France

**Keywords:** Vagus nerve, Cholinergic anti-inflammatory pathway, Vagus nerve stimulation, Rheumatoid arthritis, Inflammatory bowel diseases

## Abstract

The vagus nerve has an anti-inflammatory effect through the inflammatory reflex, which inhibits the release of proinflammatory cytokines by macrophages. Recent pilot clinical trials, using implantable bioelectronic devices, have demonstrated the efficacy of vagus nerve stimulation in adult patients with rheumatoid arthritis and inflammatory bowel diseases as an alternative to drugs, which are not devoid of side effects and are costly. In this issue of Bioelectronic Medicine, Peterson et al. report the safety of novel implantable neuroimmune modulation device for treating rheumatoid arthritis (The RESET RA study), which I will discuss in this commentary.

Rheumatoid arthritis (RA) is a chronic inflammatory disease involving mainly the joints but also extra-articular manifestations, such as rheumatoid nodules, pulmonary involvement or vasculitis, and systemic comorbidities, with a significant impact on the quality of life of patients (Smolen et al. 2016). RA has an incidence of 0·5–1% with an onset between the ages of 30 and 50 years and a woman predominance. RA probably arises from multiple hits, whereby an initial combination of environmental, with a considerable interest in the effect of the microbiome on disease risk and progression, lifestyle, and stochastic insults occurring in a genetically predisposed, epigenetically modified individual leads to breach of immunological tolerance (Smolen et al. 2016). Thus, RA has similitude with the pathogeny of inflammatory bowel diseases (IBD), such as Crohn’s disease (CD) and ulcerative colitis and can be associated (Torres et al. 2017). The reversal of inflammation is the major therapeutic target of RA thus requiring a strategic approach where regular assessment of disease activity drives therapeutic adaptations or changes of drugs i.e. the “treat to target” strategy, also recommended in the management of IBD (Turner et al. 2021). Disease-modifying antirheumatic drugs (DMARDs) target inflammation and must reduce structural damage progression. Synthetic (methotrexate, leflunomide) and biological DMARDs (TNF inhibitors, interleukin 6-receptor inhibitor, T-cell co-stimulation blockade, B-cell depletion, and janus kinase inhibitors) (Smolen et al. 2016). These drugs might mediate their efficacy by interfering with a common final pathway—namely, proinflammatory cytokine production. All biological DMARDs exhibit enhanced efficacy when combined with methotrexate and presumably any other conventional synthetic DMARD. The biological agents and the targeted synthetic DMARDs induce more adverse events than do conventional synthetic DMARDs (Smolen et al. 2016). In particular, the incidence of serious infections is increased, although it decreases over time. However, although stringent remission, or at least low disease activity, is today’s therapeutic goal for RA, many patients do not reach this target or achieve it but remain dependent on medication, implying that new therapies are still needed. In addition, many patients lose responsiveness over time, the reasons for which are unknown but might include immunogenicity, or non-adherence. Up to 50% of patients discontinue their therapy after 2 years due to lack of efficacy, toxicity, and poor compliance (Ebina et al. 2019). Results from a real-world survey reported that three-fourths of RA patients are not satisfied with treatments (Radawski et al. 2019). Patients continued to experience bothersome symptoms that affected their daily activities and life. Early diagnosis and initiation of DMARD therapy are pivotal to prevent damage from occurring or becoming clinically significant. RA has an economic burden since in the US, the estimated annual direct health care costs for RA resulted in total incremental costs of $22.3 billion (Kawatkar et al. 2012). Drug costs compromised the main component (up to 87%) of the direct costs of RA care (Hsieh et al. 2020).

Consequently, a non-drug therapy targeting anti-inflammatory pathways (i.e. anti-cytokine therapy) with no side effects and a lower cost is mandatory (Bonaz et al. 2021). Based on its anti-inflammatory properties, the vagus nerve (VN) is a prime candidate to target. Indeed, the VN activates the HPA axis through its afferents, and the cholinergic anti-inflammatory pathway, a vago-vagal anti-inflammatory reflex, through its efferents. This inflammatory reflex inhibits the release of pro-inflammatory cytokines (TNF, IL-1, IL-6) through an interaction of acetylcholine on alpha7nicotinic acetylcholine receptor of macrophages (Pavlov et al. 2018). Stimulating the VN through VN stimulation (VNS), i.e. Bioelectronic Medicine, is based on neuromodulation of the nervous system restoring organ functions and health with potentially less adverse effects than drugs (Olofsson and Tracey 2017).

The VN may be stimulated invasively at the cervical level, through a spiral electrode wrapped around the left VN in the neck. The connected cable is tunneled subcutaneously to and connected with a pulse generator placed in a subcutaneous pocket in the left chest wall, under the left clavicle (Reid 1990). The duration of the surgical implantation is ∼ 1 h. The current device is manufactured by Livanova (London, UK). Such an invasive VNS, approved by the FDA in the treatment of drug refractory epilepsy and depression in 1997 and 2005 respectively, has also been used with efficacy and no major side effects in pilot studies for the treatment of patients with RA (Koopman et al. 2016a) and CD (Bonaz et al. 2016; Sinniger et al. 2020, D’Haens et al. 2023). The three critical parameter settings of VNS are pulse width, frequency, and current intensity. VNS parameters in epilepsy and depression are current intensity 0.25 to 3.5 mA, pulse width 250–500 µs, stimulus frequency 20 to 30 Hz, duty cycles of 30 s ON and 5 min OFF (Bonaz et al. 2013). High-frequency (20–30 Hz) stimulation is generally used to activate vagal afferents for the use of VNS in epilepsy and depression while it is thought that, in animal models, low frequency (1–10 Hz) stimulation activates preferentially vagal efferents and consequently the cholinergic anti-inflammatory pathway (Borovikova et al. 2000; Bernik et al. 2002). There is a frequency/dose-effect of VNS on acute blood flow changes. Compared to 5 Hz, 20 Hz VNS produced more acute brain activity changes (Lomarev et al. 2002). However, even a very low frequency of stimulation of 1 Hz activates brain loci (Osharina et al. 2006). A low frequency of stimulation of 5 Hz also activates vagal afferents (Reyt et al. 2010). In addition, both stimulation of afferents and efferents is of interest in the anti-inflammatory properties of VNS (Bonaz et al. 2021). However, the optimal parameters of VNS to achieve efficacious inflammation-related symptomatic relief by recruiting the appropriate fibers within the VN are still unknown. Tsaava et al. (2020) reported in experimental conditions that electrical stimulation parameter selection is critically important for the modulation of cytokines via the cervical VN and that specific cytokines can be increased by electrical stimulation in the absence of inflammation. In addition, the periodicity and duration of VNS is a matter of question between a continuous ON-OFF stimulation (Bonaz et al. 2016; Sinniger et al. 2020) and an electrical stimulation restricted to 1–4 times daily in sessions lasting 1–5 min (Koopman et al. 2016a; D’Haens et al. 2023), thus potentially limiting off-target effects such as hoarseness and discomfort and also saving battery power. The electrode used for classical invasive VNS does not stimulate all the VN and may stimulate non-appropriated VN fibers thus resulting in off-target effects. Indeed, the VN was not completely encircled by the electrode (Bonaz et al. 2016; Sinniger et al. 2020; D’Haens et al., 2023) and fibres not covered should require higher stimulation, whereas fibres located near the perineurium of a fascicle were exposed to a stronger electrical field (Helmers et al. 2012). Moreover, anatomical variations of the cervical VN can affect the responses of nerve fibres to electrical signals delivered through an electrode (Pelot et al. 2020). Selective VNS, such as fibre-selective or spatially-selective VNS, aims to mitigate this by targeting specific fibre types within the nerve to produce functionally specific effects (Fitchett et al. 2021). Finally, the cost of invasive VNS ranges from USD 30,000 to USD 50,000 (Badran et al. 2018). Common side effects reported for VNS of afferents (20–30 Hz) in epilepsy and depression are cough, hoarseness, voice alteration, and paresthesias. They are generally well tolerated and usually related to the ‘on’ phase of stimulation, often fade with time and easy to control by reducing the stimulation intensity (Ben-Menachem 2001).

In this issue of Bioelectronic Medicine, Peterson et al. (2024) evaluated the safety of an active implantable device for treating RA. The RESET-RA study is a randomized, double-blind, sham-controlled, multi-center, two-stage pivotal trial that enrolled patients with moderate-to-severe RA with incomplete response or intolerance to at least one biologic or targeted synthetic DMARDs. The originality of this study is to use a neuroimmune modulation device from SetPoint Medical (Valencia, CA) which was implanted on the left cervical VN within the carotid sheath in all patients. In contrast to the device from Livanova classically used in the treatment of epilepsy, and the pilot studies in CD and RA, the device of Setpoint is composed of 2 implanted components: a miniaturized pulse generator with integrated electrodes and a silicon pod that positions the pulse generator on the left VN; and two external components: the wireless charger and an iPad application for programming the pulse generator. The stimulation parameters (10 Hz pulse frequency, 250 µs pulse width, 60-second pulse train duration, once/day) were based on extensive preclinical work in animal models of inflammation (Levine et al. 2020) and specifically designed to activate the inflammatory reflex to decrease systemic inflammation.

Not yet evaluated, the efficacy primary endpoint is after 12 weeks, and patients are then to be followed across a long-term extension period (180 weeks). The study plans to enroll up to 250 patients across two consecutive stages at up to 45 clinical study sites. In their manuscript, the authors focus on the Stage 1 of the study that included demographics, enrollment rates, device implantation rates, and safety of the surgical procedure, device, and stimulation.

No intraoperative complications, infections, or surgical revisions were reported, as well as unanticipated adverse events during the perioperative period and at the end of Stage 1. There was no study discontinuations due to adverse events, as well as no serious adverse events related to the device or stimulation. Two serious adverse events, vocal cord paresis and prolonged hoarseness, were reported in 2 patients, classically known as complications of surgical implantation with VNS devices. Vocal cord paresis resolved after vocal cord augmentation injections with filler and speech therapy, and prolonged hoarseness improved with speech therapy but mild hoarseness persisted.

The interest of this study is the device, which is an innovative neurostimulation system with a miniaturized active implanted device with integrated electrodes, so that there is no need to tunnel the electrode until the upper part of the chest. If the technique used to isolate the nerve is similar to implantation of other device, with electrode and separate stimulator such as the one from Livanova, the pulse generator used in the RESET-RA study has a significantly smaller form and integrated electrodes that obviate the need to tunnel electrical leads from the target stimulation site in the neck to a separate pulse generator in the chest. Furthermore, the device minimizes manipulation of the nerve as the electrodes are not required to be coiled around the nerve, and there are no tethered leads. The longer battery life of at least ten years for the study’s pulse generator is another advantage compared to other traditional implanted VNS devices, which have batteries that last 3 to 8 years. In addition, the use of a low frequency stimulation, i.e. 10 Hz, instead of the 20–30 Hz frequency performed in epilepsy, add for the duration of the battery. The chronic mechanical safety of the device was previously tested experimentally in animals. Blood flow through the major cervical vessels was unaffected, and pathologic and histologic findings included normal foreign body encapsulation and an absence of demyelination and nerve damage (Levine et al. 2018). The device can be recharged externally, thus making the treatment more convenient for patients and avoiding the burden of frequent surgical procedures. Patients wore the wireless charger around their neck during programming to enable telemetry with their pulse generator through the charger and the iPad-based programming app. Once the app is connected to the pulse generator, an analysis was performed to assess current prescription settings, the total number of doses delivered, the number of doses missed, the battery level of the implant, and the impedance level. The VNS settings can be adapted to each patient, adjusting stimulation parameters such as pulse width, frequency, and current intensity to optimize the therapeutic response. In their study, electrical stimulation was restricted to 1 min per day, thus potentially limiting off-target effects such as hoarseness and discomfort but also saving battery power. Stimulation for 1 min daily in the rat collagen–induced arthritis model was sufficient to inhibit disease (Levine et al. 2014). In contrast, a continuous ON-OFF stimulation (Bonaz et al. 2016; Sinniger et al. 2020) or restricted to 1–4 times daily in sessions lasting 1–5 min (D’Haens et al. 2023; Koopman et al. 2016a) was used in CD and RA.

The set-up of the study is also of interest since it is a randomized, double-blind, sham-controlled, multicenter study, while many VNS studies in chronic inflammatory disorders were only pilot studies without any control group. Sham control is difficult to achieve because choosing even a very low frequency of stimulation (1 Hz), as a control, is able to activate the brain. Consequently, in the present study, the patients were implanted but not stimulated. However, when the device is in the ON phase, patients generally perceive the stimulation. It will be interesting to see if any formal analyses of patient perception of treatment show that the blinding strategies employed were successful. The other question is whether a 1-min stimulation is able to have anti-inflammatory effects, and what is the optimal stimulation mode (duration, periodicity) to alleviate inflammation.

To my knowledge, the authors did not record heart rate variability in basal condition and at the end of Stage 1. Indeed, there is a dysautonomia, with a low vagal tone, in chronic inflammatory disorders such as IBD (Pellissier et al. 2014) and RA (Rassmussen et al. 2018). A low vagal tone can be considered a pro-inflammatory condition since there is an inverse association between vagal tone and TNF blood level in CD patients (Pellissier et al. 2014). In addition, autonomic dysfunction precedes the development of RA (Koopman et al., 2016b). Indeed, a lower parasympathetic activity, and a decreased expression level of the parasympathetic α7nicotinic acetylcholine receptor on peripheral blood monocytes, and higher sympathetic hormone (norepinephrine) were reported in patients at risk to develop RA. In addition, VNS restores vagal tone as reported in CD (Bonaz et al. 2013; Sinniger et al. 2020). Thus, recording heart rate variability is of interest when performing VNS since including RA patients with a low vagal tone could be predictive of response. Indeed, predictors of response to VNS are of interest in both RA and IBD.

Generally, VNS is performed on the left VN, which innervates the atrioventricular node, regulating the force of contraction of the heart muscle with less influence over heart rate (Bonaz et al. 2013). No significant impact on heart rate was identified in their study. VNS is a slow active therapy since its benefits increase over time, with long-term treatment associated with further improvements in seizure control (Elliott et al. 2011), and in CD patients (Sinniger et al. 2020).

Non-invasive VNS, not requiring surgical implantation of the device is an alternative to invasive VNS, since surgery may be a limiting factor for patients. Transcutaneous auricular (ta) VNS is based on the stimulation of the cymba concha of the ear, reported to be 100% innervated by the auricular branch of the VN (ABVN; Peuker and Filler 2002), which is afferent to the nucleus tractus solitarius, the entry point of the VN into the central nervous system. However, the innervation of the concha is complicated by multiple neural communications of partly somatogenic and branchiogenic origin: the ABVN, the auriculotemporal nerve (a sensory branch of the posterior division of the mandibular division of the trigeminal nerve), the facial nerve, the greater auricular nerve and the lesser occipital nerve (Butt et al. 2020). Thus, a clear consensus on the auricular sites that are most densely innervated by the ABVN and whether the brain regions secondarily activated by electrical auricular VNS depend on specific parameters has yet to be achieved. Neurostimulation of the concha activates the same brain loci than cervical invasive VNS and stimulate the inflammatory reflex. Preliminary results provide support for its anti-inflammatory effect in patients with RA (Marsal et al. 2021), as well as in pediatric IBD patients (Sahn et al. 2023). However, in a very recent randomized, double-blind, sham-controlled trial, auricular VNS did not meaningfully improve RA disease activity (Baker et al. 2023). The only side effects are related to the administration of transcutaneous electrical current, which causes redness and skin irritation in some individuals at the site of stimulation (Redgrave et al. 2018). One limit of non-invasive VNS is the compliance to the treatment, which is not the case with implanted devices. In addition, transcutaneous stimulation is inherently non-specific, activates a different group of fibers than implanted VNS, and that may not provide all the same effects as implanted VNS, although invasive VNS may also have off-target effects. Only positive data from sizable double-blind studies should determine the applicability of VNS or non-invasive VNS to disease.

In conclusion, neuroimmune modulation of RA with this novel miniaturized device was safe, well tolerated and should be cost-effective compared to biologic DMARD therapy. The clinical efficacy results of the fully completed study are awaited with interest.

## Data Availability

Not applicable

## Abbreviations

ABVN: Auricular branch of the vagus nerve
CD: Crohn’s disease
DMARDs: Disease-modifying antirheumatic drugs
IBD: Inflammatory bowel diseases
RA: Rheumatoid arthritis
taVNS: Transcutaneous auricular vagus nerve stimulation
VN: Vagus nerve
VNS: Vagus nerve stimulation
